# Cystic duct remnant: a rare cause for post-cholecystectomy syndrome

**DOI:** 10.1259/bjrcr.20170043

**Published:** 2017-11-22

**Authors:** Priyank S Chatra

**Affiliations:** Department of Medical Imaging, Geraldton Regional Hospital, Geraldton, WA, Australia

## Abstract

Post-cholecystectomy syndrome is a group of disorders resulting from complications of laparoscopic cholecystectomy. Here we report a case of a 59-year-old female patient with history of cholecystectomy who presented with right hypochondrium pain and bloating. Ultrasound and magnetic resonance cholangiopancreatogram revealed a tubular cystic lesion with calculus *in situ* in the gallbladder fossa in keeping with cystic duct remnant.

## Case report

A 59-year-old female patient presented with abdominal pain and bloating sensation after every meal. At times, pain was severe and was localized to the right hypochondrium. The patient had undergone laparoscopic cholecystectomy for similar complaints 6 months back. On clinical examination, there was positive Murphy’s sign. Laboratory investigations revealed normal bilirubin levels.

**Figure 1.**
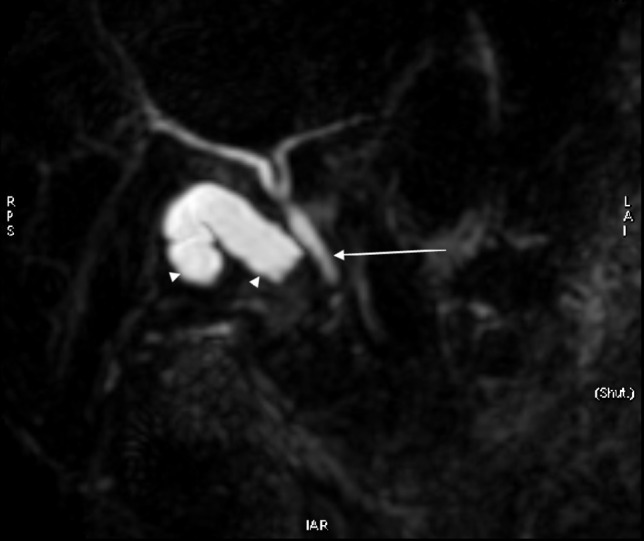
3D MRCP image showing dilated cystic duct (arrow head) and a normal appearing CBD (arrow). CBD, common biliary duct; MRCP, magnetic resonance cholangio pancreatogram.

**Figure 2.**
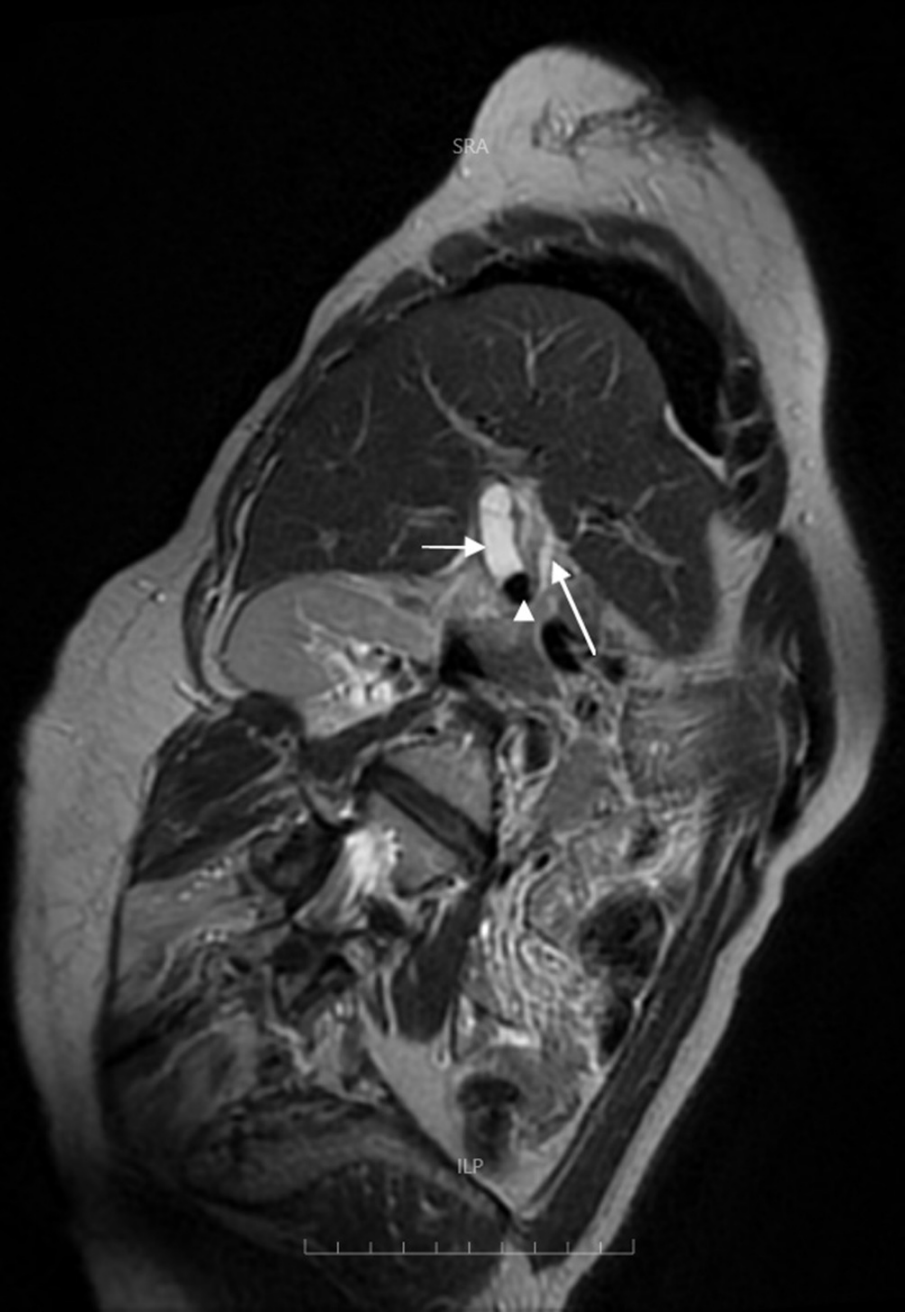
*T*_2_ Oblique coronal image showing dilated cystic duct (short arrow) with a calculus *in situ* (arrow head) and a normal appearing CBD (arrow). CBD, common biliary duct.

**Figure 3.**
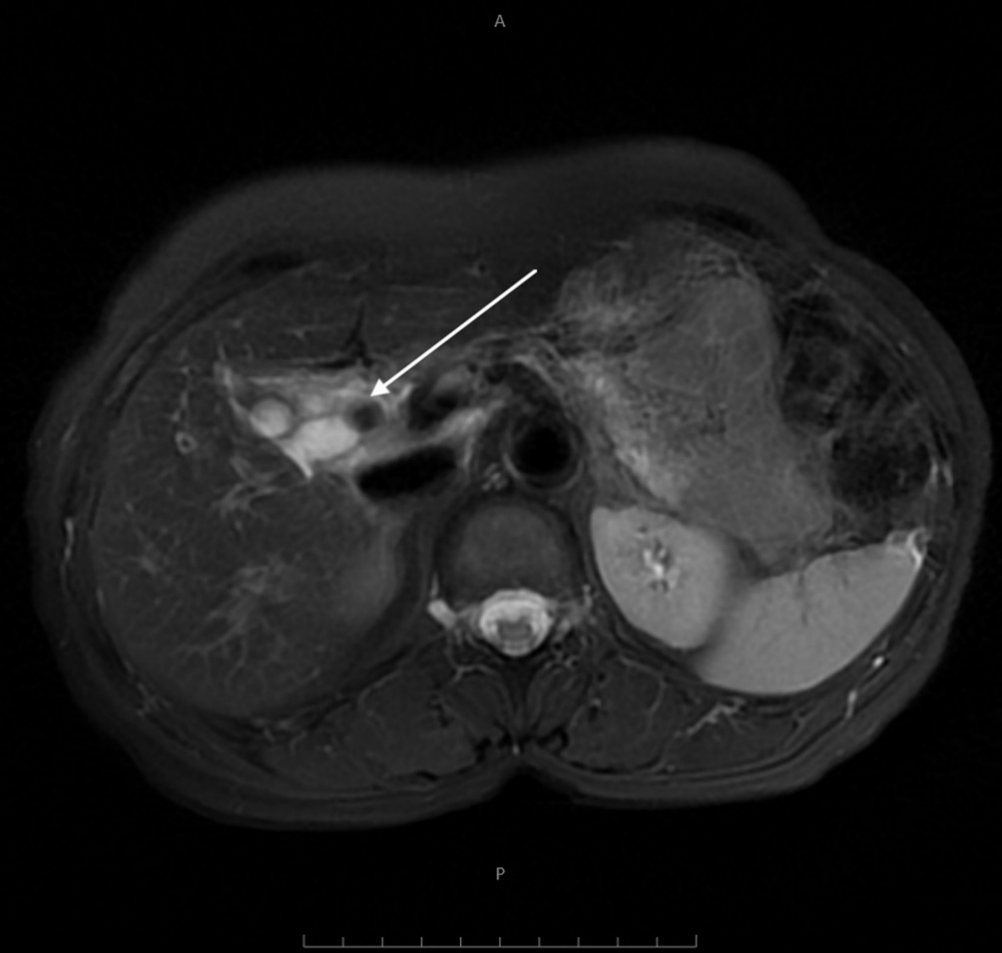
*T*_2_ axial image showing dilated cystic duct with partially visualized calculus (arrow).

Ultrasound examination of abdomen done elsewhere revealed cystic lesion in the right hypochondrium in the gall bladder bed. magnetic resonance cholangiopancreatogram (MRCP) of the abdomen suggested for better evaluation of the cystic lesion. On MRCP (Figure 1), there is an oblong cystic lesion seen in the gall bladder fossa. This cystic lesion is showing a focal *T*_2_* shortening which is interpreted as a remnant stone (Figures 2,3). The lesion is not connecting with the common biliary duct (CBD). Both the CBD and intrahepatic biliary radicles are otherwise unremarkable. Collectively, the MRCP is interpreted as remnant cystic duct with a calculus *in situ*.

Based on the above findings, the patient was subjected to laparoscopic exploration and the residual cystic duct with calculus were successfully removed. Following surgery, the patient improved dramatically with alleviation of cholestatic symptoms and follow-up ultrasound abdomen every 6 months was unremarkable.

## Discussion

Post-cholecystectomy syndrome (PCS) comprises of various conditions which present as recurrence of symptoms experienced before cholecystectomy. Onset of illness can be as short as 2 days and long as 25 years. The disorder is more common in females.^1^ These disorders can be classified as extra biliary and biliary. Majority of the causes are attributed to extra biliary causes such as peptic ulcer disease, reflux esophagitis, chronic pancreatitis, hepatitis, diverticulitis, mesenteric ischaemia and irritable bowel syndrome. Biliary causes include biliary stricture, biliary leakage, retained or recurrent biliary calculi, cystic duct remnant, dyskinesia of sphincter of Oddi and neuroma in the surgical bed.^2^

Extra biliary causes of PCS should be considered seriously as most of them can be controlled on medications. These needs to be ruled out before surgery for a suspected gall bladder disease at the first place. These conditions are often elusive but can be suspected if no calculi or gall bladder abnormality is found on cholecystectomy.^3^

Biliary causes PCS can be further classified as early and late PCS based on onset of symptoms within 2 years. Early PCS symptoms are likely due to complication of surgery such as cystic duct remnant/CBD calculus, bile duct injury or leak. Late PCS include biliary stricture, recurrent CBD stone, cystic duct remnant stone/ inflammation. Other late causes include papillary stenosis and biliary dyskinesia.^3^

Cystic duct remnant is defined as duct remnant more than 1 cm with or without calculus causing PCS.^4^ Cystic duct remnant is itself may be a cause for PCS. Remnant gall bladder with a cystic duct stump is also not an uncommon cause for PCS. Such gall bladder remnants are invariably left behind from subtotal cholecystectomy. Subtotal cholecystectomy is done when a complicated calot’s triangle anatomy is encountered. Other indications include gall bladder disease in portal hypertension and Mirizzi’s syndrome.^5^

 Calculus in the cystic duct remnant/CBD can be retained or recurrent. Retained calculus are left behind on surgery whereas recurrent calculi are formed because of biliary stasis. This is more common in the gall bladder remnant with a cystic duct stump.^6^ Biliary causes of PCS have increased over a few decades after the advent of laparoscopic cholecystectomy. This is because of the practice of ligating the cystic duct close the gall bladder to avoid CBD injury. In open cholecystectomy, the cystic duct is ligated as close to the CBD as possible.^7^ Common reason for leaving a long cystic duct remnant is failure to identify the gall bladder-cystic junction. This is even more common in patients with acute cholecystitis.^8^

**Figure 4.**
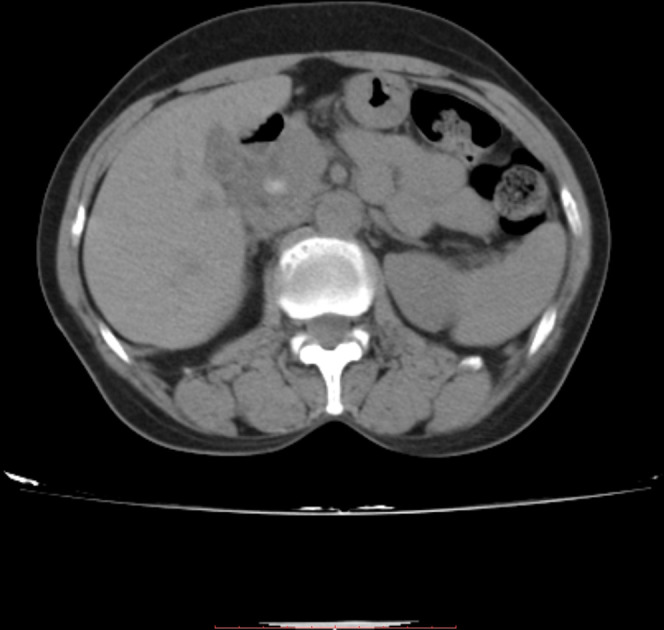
Non-contrast CT axial image showing calcified cystic duct calculus.

**Figure 5.**
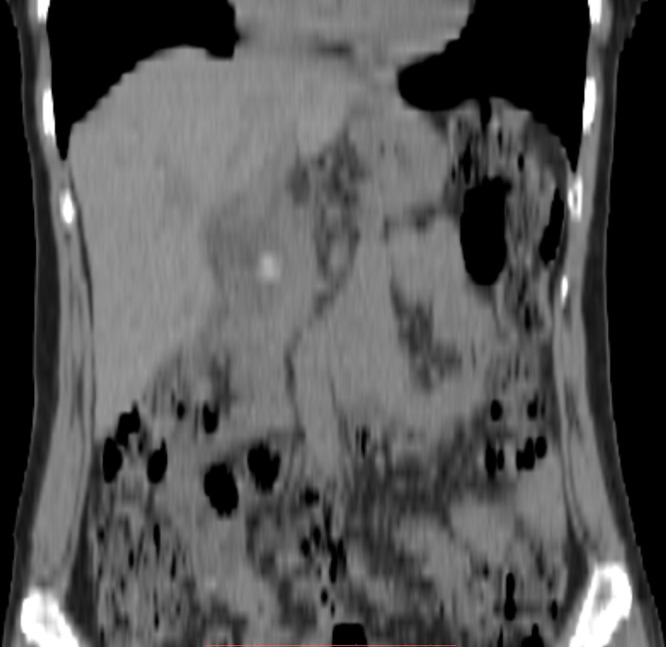
Non-contrast CT coronal reformatted image showing calcified cystic duct calculus.

Detection of the cystic duct remnant is mostly done with imaging. Imaging modalities available include ultrasound, endoscopic ultrasound, endoscopic retrograde cholangiopancreatography (ERCP), CT and MRCP. Ultrasound is the first and foremost investigation available. Accuracy of 60% was found in one of the studies towards detecting cystic duct remnant.^8^ However, the modality is user dependent. Endoscopic ultrasound has been proposed to reduce the number of ERCP examination in PCS patients by about 50%^9^ although lack of expertise is a major drawback. Preoperative ERCP is done to look for CBD stone as a cause for PRS. It's also used in cases when on-table cholangiogram has shown a spilled gall stone with further therapeutic options. ERCP and stenting is also useful in cases of biliary stricture causing PRS.^1^ On-table cholangiogram is now done in most centres before lap cholecystectomy to look for any gall stone spillage or undiagnosed CBD stones.^1^ CT is useful to detect calcified cystic duct remnant stones as in our case (Figures 4, 5), but further information cannot be derived. MRCP is the modality of choice for detecting cystic duct remnant.^3^ It appears as an oblong fluid collection in the gall bladder fossa. For an untrained eye, it can be easily confused to a normal gall bladder. However, this oblong fluid collection shows no continuity to the CBD which is best demonstrated on MRCP images. Differential for such an imaging finding could also include a biloma. Presence of calculus in the oblong fluid collection firmly favours the former.

Treatment of the cystic duct remnant was traditionally done as open technique as laparoscopy was thought to be risky due to scaring of the surgical bed. Off late, due to expertise in laparoscopic surgery, minimal access surgery is now considered the management of choice.^8^ The re-exploration rates in lap and open cholecystectomy were found similar in the latest study by Sanjay Kumar Saroj et al.^10^ Other treatments reported include ERCP with basket, laser lithotripsy, ESWL with or without endoscopic stone removal.^11^ These treatments are mostly reserved for patients unfit for surgery and are also limited by the availability of expertise.

## Conclusions

Careful surgery, sticking to the basics helps in reducing possibility of cystic duct remnant leading to PCS. Careful evaluation is needed to rule out all non-biliary causes before pin-pointing the cause for PCS. MRCP is the modality of choice to look for biliary cause of PCS and should be interpreted with utmost care looking for all possible complications.

## Learning points

1. Failure to identify the gall bladder-cystic junction is the foremost cause for leaving a long cystic duct remnant.
2. Careful surgery, sticking to the basics helps in reducing possibility of cystic duct remnant leading to post-cholecystectomy syndrome.
3. Cystic duct remnant can be easily confused to a normal gall bladder. However, absence of continuity to the common biliary duct on magnetic resonance cholangiopancreatogram images is a key finding differentiating the former from the latter. Other differential for this finding is a biloma. Presence of calculus in the oblong fluid collection favours the former.

## Consent

Written informed consent for the case to be published (including images, case history and data) was obtained from the patient(s) for publication of this case report, including accompanying images. 
